# “This path I must walk alone”. Challenges experienced by older patients while recovering from severe COVID-19 – a qualitative study

**DOI:** 10.1186/s12877-022-02959-y

**Published:** 2022-03-28

**Authors:** Kristi Elisabeth Heiberg, Nina Jøranson, Anne Kari Tolo Heggestad, Grete Breievne, Hilde Lausund, Marius Myrstad, Anette Hylen Ranhoff, Marte Meyer Walle-Hansen, Vigdis Bruun-Olsen

**Affiliations:** 1 Department of Medical Research, Clinic of Bærum Hospital, Vestre Viken Hospital Trust, Drammen, Norway; 2 Institute of Physiotherapy, Department of Health Science, OsloMet - Oslo Metropolitan University, Oslo, Norway; 3 VID Specialized University, Oslo, Norway; 4 University of South-Eastern Norway, Drammen, Norway; 5 Diakonhjemmet Hospital Trust, and University of Bergen, Bergen, Norway

**Keywords:** COVID-19, Older patient, Experiences, Recovery, Rehabilitation, Qualitative

## Abstract

**Background:**

In March 2020, the COVID-19 pandemic challenged both the Norwegian population and healthcare system. In this study we explored how older men and women experienced rehabilitation and recovery after hospitalisation due to severe COVID-19.

**Methods:**

Semi-structured interviews with 17 participants aged 60–96 years were performed 6 months after discharge from hospital. A thematic descriptive analysis was conducted.

**Results:**

The results revealed that the participants experienced a challenging span between loneliness and companionship in recovering from severe COVID-19. The four subthemes highlighted experiences of being discharged to home and left to themselves, the importance of exercise and companionship at rehabilitation stay, requirement of self-effort and time to recover, and the challenging span between loneliness and companionship when being with family.

**Conclusion:**

Among participants, the experiences of loneliness throughout the recovery period were striking. An individualised approach including psychological support should be emphasized in primary healthcare to promote recovery in older survivors after severe COVID-19 and their next-of-kin.

## Background

During the last weeks of 2019 the first cases of novel coronavirus 2019 (COVID-19) were confirmed in China [1]. The virus affected individuals at varying degrees, from a few days of mild symptoms to death [2]. Older people were especially vulnerable to the infection. Many of the oldest have faced death or serious illness with hospitalisation for a varying number of days [3]. Six months after the outbreak of the pandemic the survivors were in different stages of their recovery process.

Following critical illness and hospitalisation, it is common, especially for older people, to experience loss of physical function [4] and a need for rehabilitation to fully recover. In the early days of the pandemic there were no clear guidelines as to whether the patients would need a rehabilitation stay or could be sent directly home after hospitalisation. Based on clinical reasoning it became common practice that some patients were discharged to home while others, who had been on a mechanical ventilator for several days and had a longer hospital stay, received interdisciplinary rehabilitation follow-up. However, it was unclear which type of rehabilitation program the patients would need. Moreover, it was still unknown how and to what extent the patients would fully recover [5].

It has been suggested that the pandemic might influence health-related quality of life (HR-QoL) in general, and that older people had a higher risk of experiencing reduced HR-QoL after the disease than younger [6]. Among hospitalised patients, studies have shown that many have reported impaired health status and reduced quality of life three [7] and 6 months [3] after discharge. This suggested that they suffered from the post COVID-19 condition [8]. In these studies, including a previous systematic literature review [9], recovery outcomes following COVID-19 were measured as endpoints [3, 7, 9]. Few studies have explored the patients’ own experiences of going through long-term recovery after COVID-19. In one study the patients were interviewed about psychological disturbances throughout the disease crises. They reported living “in limbo”, psychological distress, and the burden of being an infectious carrier [10]. In another study, 11 patients were interviewed during hospitalisation about their experiences of being in isolation and their anticipated post-discharge life [11]. The patients conveyed both positive and negative experiences of the infection, isolation, and illness. Interestingly, all the patients expected to make a full recovery. To the best of our knowledge, no previous studies have explored the challenges older patients have experienced from rehabilitation, homecoming, and being in long-time recovery after COVID-19.

Insight into the patients’ experiences of challenges in the recovery process will give a broader picture of which factors that have contributed to recover from the disease. This knowledge is important to healthcare professionals to enable them to improve and individualise their support to patients recovering from severe COVID-19. The aim of this study was to explore how older patients who were hospitalised due to severe COVID-19 in the early outbreak of the pandemic, experienced their rehabilitation and recovery process during the first 6 months.

## Methods

### Design

The study had an explorative and descriptive design with qualitative interviews conducted in October and November 2020. It was part of a multi-centre cohort study from South-Eastern Norway.

### Participants and context

Participants, aged 60 years and older, were recruited from two of the four hospitals in the multi-centre study, as part of a follow-up consultation with a physician approximately 6 months after hospitalisation [3]. Those considered by the physicians (AHR, MMW-H) to be physically and cognitively able to be interviewed were contacted by the researchers (AKTH, NJ, HL, GB, VB-O, KEH) and asked to participate. They were selected depending on age and gender and formed a convenient sample. A written informed consent was obtained.

Fourteen in-depth interviews with 17 participants were performed. Three of the interviews were with couples in which both were hospitalised due to COVID-19 during the same time period. The participants were between 60 and 96 years old, eleven were men and six were women. They had been hospitalised for 2–61 days (mean 18 days), and approximately 50% had been treated on a mechanical ventilator for 10–55 days. Nine participants were discharged directly to home, while eight were discharged to a rehabilitation stay (Table 1).

**Table 1:** Characteristics of the participants

Participants	Age (range)	Cohabitant	Days spent on mechanical ventilator	Length of hospital stay (days)	Rehabilitation yes/no
P1	70–74	yes	31	42	Yes
P2	75–79	yes		22	Yes
P3	95–99	no		14	No
P4	60–64	yes	14	21	No
P5	75–79	no		3	No
P6	60–64	yes		3	No
P7	70–74	no		21	No
P8	60–64	yes		34	Yes
P9	65–69	yes		5	No
P10	70–74	yes		10	No
P11	70–74	yes	10	28	No
P12	65–69	yes		2	Yes
P13	85–89	yes		3	Yes
P14	90–94	yes		4	Yes
P15	80–84	yes	12	19	Yes
P16	80–84	yes	55	62	Yes
P17	65–69	yes		9	No

### Data collection

An interview guide was prepared beforehand and contained questions about the participants’ experiences from their rehabilitation and recovery process 6 months after hospitalisation. The prepared questions were open-ended and aimed to produce as rich descriptions as possible. Examples of questions posed were: “Can you describe your recovery process after hospitalisation?” “Can you describe your need for rehabilitation?” “Please describe your experiences from the rehabilitation stay.” “Please describe facilitators and barriers in your recovery process.” Encouraging prompts were made throughout, such as “What do you think about this?” or “Could you please elaborate?” Six researchers performed the interviews, working in pairs (AKTH/NJ, HL/GB, VB-O/KEH). They were female nurses or physiotherapists and had broad experience from clinical work and qualitative research.

The participants could choose to be interviewed either at home or at the hospitals’ research department. Each interview lasted for 45–90 min. The interviews were audio recorded and externally transcribed into text.

### Data analysis

Data analysis was performed according to a method of thematic descriptive analysis inspired by Braun and Clarke [12–14] Thematic analysis is described as an independent qualitative descriptive approach for identifying, analysing, and reporting patterns (themes) within textual data [15]. Furthermore, it organises and describes the data set in fairly rich detail [12].

Thematic analysis can be either an essentialist or a realist method [12]. Presently, the realist method was applied, which reported experiences, meanings, and the reality of participants. In the analytic process, the six steps according to Braun and Clarke (2006) were followed [12]. These steps were: 1. Familiarising with the data, 2. Generating initial codes, 3. Searching for themes, 4. Reviewing themes, 5. Defining and naming themes, and 6. Producing the report [12]. In the analytic process the researchers were going back and forth between the steps described above to accomplish deep reflection and engagement with the data [13].

The text was very detailed and rich. First, the text was read separately several times by the six authors (KEH, VB-O, NJ, AKTH, GB, HL) to obtain a global sense of the participants’ experiences. They agreed on the main essence of the data. For the present study, the guiding question was: What could these data tell us about the participants’ rehabilitation and recovery process following COVID-19? Following the initial reading, two of the authors (KEH and VB-O) performed the last part of the analysis. They read through the data several times to identify places in the text that were related to the participants’ experiences of the recovery process. Excerpts of the text were identified and coded manually, that is, the meaning of each excerpt was condensed into codes. Then the codes were combined and grouped into initial subthemes. After going back and forth between the analytic steps we considered combination of codes to be grouped into initial sub-themes. There was a constant interplay in the entire process between the various analytical steps. A consensus between the two main authors (KEH and VB-O) was reached through discussions. The analytic process produced four final sub-themes, describing one main theme (Table 2).

**Table 2:** Examples from the analytic process

Excerpts	Codes	Subthemes	Main theme
*-There was no follow-up. (Male participant, P3)*	No follow-up	Discharged to home and left to themselves	Recovering from COVID-19 – a challenging span between loneliness and companionship
*-The corona team called every evening to hear if I had … well …*. *I do not exactly know what they wanted … It seemed like they were afraid I would fall?? (Female participant, P9)*	Weird and unintentional follow-up		
*-I realized that if I couldn’t manage to walk again, it would seriously impact future life. The horrible thought of being tied to a wheelchair, and the importance of being able to stand on my own feet … My goal could be expressed in one single sentence: The rehabilitation program was going to help me to walk again. (Male participant, P11)*	The importance of exercise	Discharged to rehabilitation stay – from isolation and loneliness to exercise and companionship	
*-But it’s quite clear that after being bedridden for so long, exercising is important to regain physical function. If you’re not physical fit, normal life becomes difficult. (Male participant, P15)*			
*-Actually, I guess one of the most important things was the companionship. I was desperate to talk to someone, and at rehab there were like-minded people. (Male participant, P1)*	The importance of being together		

In qualitative research, it is a key aspect to make the authors’ preconceptions visible [16]. The two main authors are physiotherapists having a strong belief that exercise are essential to recover from serious disease. These preconceptions were challenged by some of the interviewees’ expressions, which enabled us to discuss our preconceptions throughout the analytic process.

### Ethics

The multi-centre cohort-study was planned in the beginning of the pandemic as an observational study [17]. It aimed to investigate recovery outcomes in older people with COVID-19 in several EU countries and Norway. The Norwegian Geriatric Society approved the study, and ethical approval was granted by the Regional Research Committee in Eastern Norway (reference number 155425). In the present sub-study, only participants who gave an informed written consent to be interviewed were included. They were assured confidentiality and informed that they could withdraw at any time without consequences.

## Results

The participants experienced various challenges related to their recovery process after discharge from hospital. The thematic analysis yielded one main theme and three corresponding subthemes that are presented in the following sections. Excerpts from the interviews are inserted to substantiate descriptions of the subthemes and are presented in italics. Quotation marks refer to the participants presented in Table 1.

### Main theme

#### Recovering from COVID-19 – a challenging span between loneliness and companionship

The overarching challenge was the experience of being alone in various situations during the recovery process, but also the importance of companionship. Many of those who were discharged directly to their home experienced to be abandoned, while those who went to a rehabilitation stay went from isolation to being with others. The participants realized it was important to balance rest and activity, with an overarching insight that their self-effort was the crucial factor to recover. Furthermore, the participants reflected upon how their relationship with family members was a challenging span between companionship and loneliness. Being in recovery was experienced as a lonely path to walk.

#### Subthemes

##### Discharged to home and left to themselves

The participants experienced the follow-up after hospitalisation as diverse and inconsistent. They had been critically ill and bedridden, first at home before being in hospital for various length of stay. Nine of the 17 participants were discharged directly to their home. Many had no further contact neither with the hospital nor the municipality healthcare service. Follow-up was absent and they were left to themselves. Others reported that the only contact they had with healthcare professionals were regular phone calls from the municipal “corona office”. These phone calls were experienced as weird and unintentional and often led to a remote and confusing loneliness. This confusion can be illuminated by the following expression:

*The corona team called every evening to hear if I had … well …. I do not exactly know what they wanted … It seemed like they were afraid I would fall??* (Female participant, P9)

Participants who went directly to home had to recover on their own and gradually resume their usual activities and exercise regime. They were not referred to further treatment by an interdisciplinary rehabilitation team, although many still had problems with breathing and reduced physical function and fitness.

##### Discharged to rehabilitation stay – from isolation and loneliness to exercise and companionship

Those most severely ill were offered inpatient interdisciplinary rehabilitation for several weeks, also including psychological support if needed. Some participants expressed that starting with structured exercise was of great importance and had helped them in their recovery. Some, especially the youngest in their sixties and seventies, told vividly how they changed their attitude towards rehabilitation. One male participant discovered that when he was about to be discharged from hospital he had “sunk to the very bottom”. He was no longer able to stand on his own feet and realized that he needed rehabilitation:

*I realized that if I couldn’t manage to walk again, it would seriously impact future life. The horrible thought of being tied to a wheelchair, and the importance of being able to stand on my own feet … My goal could be expressed in one single sentence: The rehabilitation program was going to help me to walk again.* (Male participant, P11)

*But it’s quite clear that after being bedridden for so long, exercising is important to regain physical function. If you’re not physical fit, normal life becomes difficult.* (Male participant, P15)

In the process of restoring physical strength and fitness, some of the participants also revealed the importance of balancing rest and activity:

*Rest and activity.... balanced. If you stay inactive, you will not improve. And if you exercise too hard, you will deteriorate. So balanced, yes.* (Male participant, P17)

Other elements than exercise also had significant impact on experienced recovery. These were related to “the team spirit”, belonging to a group, and the importance of shared experiences. After being in isolation at home and during hospital stay, they expressed that being with others and sharing experiences from the disease was just as important as to exercise:

*Actually, I guess one of the most important things was the companionship. I was desperate to talk to someone, and at rehab there were like-minded people.* (Male participant, P1)

During rehabilitation they also interacted with patients having other diseases. To some, there was a relief to talk to someone not suffering from COVID-19. One participant pointed out the companionship with four women suffering from chronic obstructive pulmonary disease. They had a different view of living with reduced lung capacity without focusing on “nightmares and lack of smell and taste”.

Another participant, who was sent directly home, realized after some days that he needed more help to recover. He described how the rehabilitation stay helped him out of depression, to refocus, and to be able to move on:

*I had simply buried myself a bit. My wife had complained about it as well. But at rehab - it was wonderful. It was just the right thing for me.* (Male participant, P2)

##### To recover required self-effort and time

In general, the participants were very grateful for the help and support they had received at the hospital and rehabilitation stay. After a while, many realized that they had to manage on their own, and to recover also was depending on their motivation and self-effort:

*A significant self-effort is needed to recover. I have no further expectations from healthcare professionals. You must pull yourself together. I've got all the help I can get. I can't just give up and say: “Treat me!” The rest is self-effort.* (Male participant, P17)

Furthermore, they experienced that it took time to recover - more than they expected. Both physical fitness and cognitive function needed a long time to recuperate.

##### Being with family and friends - a challenging span between companionship and loneliness

A few of the participants experienced support from family and close friends as an important factor for their recovery process:

*Having a close relationship means very much in a situation like this. There's no doubt about that.* (Male participant, P17)

An older man found a new friend and got the COVID-19 infection while staying abroad, and they both were infected simultaneously. After hospitalisation they conducted daily walks developing a companionship, which facilitated their recovery process:

*We became good friends, actually. After I returned home from the hospital, we went for daily walks. I have met a woman, who is active and lively …. She has been of great importance for my recovery.* (Male participant, P3)

Another man highlighted the importance of the support from the wife, and they had found their coping strategies:

*I have a wife who wants me to recover. She's kicking me out. “Get out for a walk! Out! No nonsense!” I appreciate her concern. Therefore, my strategy is to use our companionship for all what it is worth. I'm working hard so we can continue to stay together.* (Male participant, P2)

However, despite having family and friends who were supportive and caring, the COVID-19 was described as a solitary disease. The participants realized that they were alone in the process. To recover implied loneliness and was experienced as a lonely path to walk. This self-insight was illuminated by one of the participants:

*I must accept that this path I must walk alone. I must process and recover on my own, without burdening my family. ... … .. I feel alone at home. At home I must watch my tongue. My family says: “I guess you talk about corona again. I can't bear to hear any more of that corona stuff.”* (Male participant, P8)

According to this statement, the relationship with family members could be experienced as difficult and challenging. Some had the impression that their family members were tired of hearing about Covid-19. This may have increased the feeling of loneliness.

Others expressed challenges related to relatives not understanding what it was like to go through COVID-19, and to struggle with long covid. Some participants expressed that they were no longer the same person, a change that was difficult for the family members to comprehend. Only those who had had the disease could fully understand what it implied. For participants, this led to a feeling of not being understood by their family members, which in turn increased their experience of loneliness.

On the other hand, COVID-19 was also challenging for the next-of-kin. They were scared and worried about their loved one’s health condition, and several of the participants expressed that their family members had been traumatised:

*My wife has had a very tough time. Especially when I was in the hospital on the mechanical ventilator. There are still ups and downs. When she sees me with a face mask, for example when we go shopping, she receives flash backs from that time. She suffers from post-traumatic stress.* (Male participant, P4)

Some couples were infected at the same time and found themselves in a double role. They watched their loved ones being seriously ill in parallel with coping with their own illness:

*We had COVID-19 at the same time, but my partner’s role has been a double role*. *Not many next-of-kin end up in such a situation. A lot of them must have had a tough time.* (Male participant, P11)

## Discussion

The results from this study indicate that participants had to overcome several challenges in recovering from COVID-19. The overarching challenge was the experience of loneliness in various situations. Loneliness was related to arbitrary follow-up after hospitalisation, their need for exercise and companionship, their experiences of loneliness in family relationships, and to the experience that recovery was dependent on their own self-effort. On the other hand, the support from the interdisciplinary team at the rehabilitation stay, the companionship with others, and the support from family members were facilitators in the struggle to recover from COVID-19.

In the early pandemic phase, the COVID-19 was a new and unknown disease. There was lack of clearly defined care pathways for patients discharged from hospital and for those with long-term persisting symptoms [18], both in Norway and other countries. Many of those critically ill and treated in the intensive care unit (ICU) were transferred from hospital to interdisciplinary rehabilitation facilities for approximately 2–8 weeks. Others were transferred home. However, our findings suggest that also those who were not treated in an intensive care unit (ICU), but with oxygen supply in hospital, expressed that they needed structured rehabilitation after discharge from hospital. In many cases they were left to themselves at home with their family. It seemed that referral to rehabilitation was deficient, and rehabilitation offered was arbitrary. One reason may be the strict infection control restrictions that led to reduction in municipal services and reduced access to rehabilitation. This may have led to increased loneliness and isolation, especially for the older patients [5]. However, in June 2021 the Norwegian Directorate of Health published “National Plan for rehabilitation after COVID-19”, suggested that rehabilitation in the municipalities should be given either in institutions, as home-based rehabilitation, or at private physical therapy institutes [19]. Hopefully, these consistent rehabilitation pathways have contributed to improve the rehabilitation offered to patients after COVID-19. There were no guidelines on what rehabilitation after COVID-19 in older survivors should contain. However, the rehabilitation was generally aiming to improve respiratory function as well as mobility and physical function in the form of exercise [20]. In the present study, the participants expressed that the companionship with others meant a lot in the recovery process. Being in a group and experiencing the “team spirit” was motivating and encouraging.

The various experiences and differing degrees of dysfunction among our participants were in line with a previous study which concluded that to optimize recovery, personalised plans should be designed according to the patients’ age, sex, lifestyle, hobbies, occupation, and physical condition [21]. It is suggested that patients with COVID-19 should be offered psychological help and support [19, 22]. In retrospect, it is clear that many of our participants needed a personalised rehabilitation plan, as also stated by Sun et al., (2020) [21]. They had been critically ill and produced insights into the need for comprehensive and individualised rehabilitation across the care continuum. Previously, it has been shown to be crucial for patients with ongoing support from primary healthcare professionals during recovery in their struggle to ease their loneliness and attain functional and meaningful lives [18, 23, 24].

Some participants reported that family members were frightened and suffered from post-traumatic stress syndrome. In line with this, prior research has shown that the mental health of family members to ICU-survivors may be affected [4]. Psychological problems created within the family, such as a cold atmosphere, have also previously been reported [10]. A multidisciplinary management plan aimed to improve the survivors’, and also their family members’, long-term functioning capacity and quality of life should be emphasized. In addition, psychological support should be prioritised by healthcare professionals [4].

### Limitations

This study has its strengths and weaknesses. To preserve variability and reflexivity and establish credibility, six of the authors (AKN, NJ, GB, HL, VB-O, KEH) read all the interviews. The two main authors (KEH and VB-O) analysed data independently and together. Consensus in the interpretation of the participants’ experiences was achieved among the two main authors, assuring dependability. The convenient sample of 17 participants, aged 60 years and older, from both genders, and who had been hospitalised with severe COVID-19 has strengthened the validity of the findings. However, the participants may not have been fully representative for the population of older patients with serious COVID-19 in Norway as 15 of them had an educational level above 12 years. According to the criteria for reporting qualitative research [25] the authors’ preconceptions, gender, and profession as physiotherapists are clarified to assure the credibility. Six researchers performed the interviews in pairs. We tried to assure that there was consistency in the data collected by following a prepared interview guide. By interviewing in pairs, we assured that no one could impose any personal biases. One weakness of the study may be that we have interviewed the participants only once after 6 months. The participants had to recall their experiences. Another limitation may be that some of the interviews were performed with couples and vulnerable information may not have been revealed.

## Conclusion

The findings from this study shed light on a disjointed multifaceted recovery path with challenges related to a lack of consistent follow-up from healthcare, and the participants’ expressed need for exercise and support throughout their recovery. In addition to exercise, companionship with like-minded and family meant a lot after being isolated for several weeks. The overarching insight into the patients’ experience of being alone even within the family is important to healthcare professionals. Individualised plans aimed to support and promote the recovery process of survivors from severe COVID-19 and their next-of-kin should be emphasized in primary healthcare.

## Data Availability

The datasets generated and analysed during the current study are not publicly available due to the limited permission given from the Regional Committee for Ethics in Medical Research (South-East Norway), but are available from the corresponding author on reasonable request.
